# Loss of Vagal Sensitivity to Cholecystokinin in Rats Born with Intrauterine Growth Retardation and Consequence on Food Intake

**DOI:** 10.3389/fendo.2017.00065

**Published:** 2017-04-10

**Authors:** Marième Ndjim, Camille Poinsignon, Patricia Parnet, Gwenola Le Dréan

**Affiliations:** ^1^UMR 1280 PHAN, INRA, Université de Nantes, Institut des Maladies de l’Appareil Digestif (IMAD), Centre de Recherche en Nutrition Humaine Ouest (CRNH Ouest), Nantes, France

**Keywords:** perinatal malnutrition, gastrointestinal peptide, nodose ganglia, satiation, CCK signaling

## Abstract

Perinatal malnutrition is associated with low birth weight and an increased risk of developing metabolic syndrome in adulthood. Modification of food intake (FI) regulation was observed in adult rats born with intrauterine growth retardation induced by maternal dietary protein restriction during gestation and maintained restricted until weaning. Gastrointestinal peptides and particularly cholecystokinin (CCK) play a major role in short-term regulation of FI by relaying digestive signals to the hindbrain *via* the vagal afferent nerve (VAN). We hypothesized that vagal sensitivity to CCK could be affected in rats suffering from undernutrition [low protein (LP)] during fetal and postnatal life, leading to an altered gut–brain communication and impacting satiation. Our aim was to study short-term FI along with signals of appetite and satiation in adult LP rats compared to control rats. The dose–response to CCK injection was investigated on FI as well as the associated signaling pathways activated in nodose ganglia. We showed that LP rats have a reduced first-meal satiety ratio after a fasting period associated to a higher postprandial plasmatic CCK release, a reduced sensitivity to CCK when injected at low concentration and a reduced presence of CCK-1 receptor in nodose ganglia. Accordingly, the lower basal and CCK-induced phosphorylation of calcium/calmodulin-dependent protein kinase in nodose ganglia of LP rats could reflect an under-expressed vanilloid family of transient receptor potential cation channels on VAN. Altogether, the present data demonstrated a reduced vagal sensitivity to CCK in LP rats at adulthood, which could contribute to deregulation of FI reported in this model.

## Introduction

Metabolic pathologies such as obesity and type 2 diabetes are a worldwide public health problem especially in Western countries. Although life style, decreased physical activity and nutritional transition are the main causes of predisposition to obesity, a large body of epidemiological studies linked a low birth weight, consequence of intrauterine growth retardation (IUGR), to a higher risk of metabolic pathologies at adulthood. It is indeed well documented that low birth-weight babies present a higher susceptibility to develop obesity, insulin resistance, cardiovascular diseases, and type 2 diabetes later in life (1–3). More generally the metabolic programming concept proposes that a deleterious nutritional environment inflicted during fetal and early postnatal life could impact long-term health (4).

Animal studies have demonstrated that perinatal undernutrition may lead to a programmed hyperphagia that in the long term led to adult obesity (5). Previous studies in our laboratory demonstrated that low-protein (LP) rats eat more of a regular chow diet from weaning to 2-month old (6, 7). At adulthood despite a higher speed of ingestion (6, 8), a delay in appearance of satiety (satiation) is still observed with more food consumed during the first meal (7). LP rats have also a greater appetite for high-energy diet at adulthood (8) and a preference for high-fat food in the offspring of undernourished dams has also been reported (9), which could contribute to an increase of the body weight during adulthood and later on to obesity.

Food intake (FI) is a highly integrated behavior that relies on complex interactions between neuronal populations located in hypothalamic nuclei, brainstem, and cerebral nuclei implicated in hedonism, motivation, and activation of the peripheral autonomous system. In the central nervous system, the arcuate nucleus contains two well-characterized neuronal populations that act with opposite effects on feeding. Anorexigenic proopiomelanocortin neurons synthetize α-melanocyte-stimulating hormone and cocaine and amphetamine-regulated peptide (CART) whereas orexigenic neurons express neuropeptide Y (NPY) and agouti-related protein (10).

Animal studies have shown that altered maternal nutrition disturbs this hypothalamic system in offspring in favor of orexigenic activity predisposing to hyperphagia (6, 11). Long-term mechanisms of FI regulation by peripheral signaling (leptin, insulin) that monitor energy stores and availability to maintain homeostasis are particularly sensitive to perinatal nutrition. In LP rats, impaired leptin and insulin signaling in arcuate nucleus has been demonstrated to contribute to hyperphagia (12). By contrast, the short-term mechanisms of FI, which regulate on one hand anticipated appetite through ghrelin action and on the other hand meal size and the inter-meal time through peptides from the digestive tract have been poorly studied in maternal food-deprived offspring.

Short-term regulation of FI is controlled by the integration of digestive signals by the vagus nerve into the nucleus of the solitary tract (NTS) in the hindbrain that initiates satiation by a vago-vagal reflex (13). Among these signals, gastrointestinal peptides and mainly cholecystokinin (CCK) are key regulator of short-term FI (14). Vagal afferent nerves (VANs) are primary target of CCK and it is now well demonstrated that they represent a major site for integration of peripheral signals controlling FI (15). Indeed, CCK-1 receptors (CCK-1R) are expressed in nodose ganglia and CCK release after a meal stimulates expression of Y2 receptor (Y2-R), which responds to the anorexigenic gut peptide YY (PYY) and the release of CART in VAN while expression of the orexigenic peptide melanin-concentrating hormone (MCH) is suppressed. By contrast, during fasting, when plasma CCK concentration is low, MCH receptor expression increases while Y2-R and CART expression is reduced (16). As a gatekeeper, CCK operates this vagal neurochemical phenotype switch according to the energy state (14). Interestingly, this normal switching between feeding and fasting states is blunted in diet-induced obesity rats as demonstrated by a loss of vagal sensitivity to leptin (17), which is known to act synergistically with CCK on VAN to potentiate the satiety effect of CCK (18).

Cholecystokinin suppression of FI involves numerous signaling pathways. In NTS neurons, CCK activity involves extracellular signal-regulated kinase (ERK) signaling cascade (19–21). In nodose neurons, CCK induces an increase in intracellular calcium mediated by members of the vanilloid family of transient receptor potential (TRPV)2–5 cation channels (22). Transient calcium signal is converted by calcium/calmodulin-dependent protein kinase (CaMKII), which autophosphorylates and functions as an intracellular signaling element. Prolonged phosphorylation of CaMKII reflects cellular activation (23).

We hypothesized that in LP rats, alteration of short-term FI and particularly satiation could be due to a loss of vagal sensitivity to CCK and/or an alteration of the vagal phenotype leading to a compromised integration of short-term satiety signals. Therefore, the aim of the present study was to investigate the reactivity to CCK in the adult offspring of dams fed a control or a LP diet during lactation and gestation. First-meal pattern following fasting was studied in physiological cages and plasma concentrations of various gastrointestinal peptides were measured in pre- and postprandial period. A dose–response to intraperitoneal (i.p.) CCK agonist (CCK-8S) injection was performed to quantify CCK sensitivity by measuring FI. Neurochemical vagal phenotype was determined in fasting/feeding states and vagal activation following CCK injection was analyzed by measuring phosphorylation of CaMKII and ERK in nodose ganglia.

## Animals and Methods

### Animals

Pregnant females (gestation day 1) were obtained from Janvier (Le Genest Saint Isle, France), housed individually under standard laboratory conditions with free access to either a control (20% w/w protein, *n* = 8) or an isocaloric LP diet (8% protein, *n* = 8) through gestation and lactation. Both diets were purchased from AB diets (Woerden, The Netherlands) and composition is provided in Table S1 in Supplementary Material. At delivery, litter size was adjusted to eight male pups per dam. Pups were pooled and randomly attributed to foster mothers to create two experimental groups. Pups born from control dams were adopted by foster control ones (control, *n* = 24) and pups born from LP dams were attributed to foster LP dams (LP, *n* = 24). Control and LP offspring were weaned at 21 days of age and received a standard laboratory chow (A04, Safe, Augy, France) until adulthood. As previously described in this model (6, 24), birth weight of LP rats was 7–9% lower than control birth weight and this difference persisted until adulthood (130–160 days).

### First-Meal Pattern Analysis

At 160 days of age, rats (8–12 animals/group) were individually housed (22 ± 2°C, 12:12-h light/dark cycle, lights on at 7:30) for 3 days in plexiglas Phecomb cages (Bioseb, Vitrol, France) equipped to monitor meal pattern by continuous and automatic weighing of food. Phecomb system weights the food tray for each second. It allows precisely quantifying the food consumption and identifying feeding events. Artifacts as large vibrations when the rat enters in contact with the tray but without eating are taken into account by filters on the hardware and the software (Phecomb system monitoring software Compulse). A percentage of reliability of the quality signal was calculated by the software and only experiments with a percentage >80% have been used. A meal was defined with a minimal size of 0.5 g and a minimum inter-meal interval of 20 min. A meal is composed of several bouts. Meal parameters extracted from Compulse software included latency to eat, meal size, duration of the meal, inter-meal interval, and satiety ratio. After 48 h of fasting and 24 h of acclimatization to the cage, data were recorded from the beginning of the second day (8:00 a.m.) each 5 s over a 24-h period. This prolonged caloric restriction of 48 h was chosen on the basis of previous reports showing that 24–48 h duration of fasting triggers VAN to switch from anorectic to orexigenic phenotype (16, 25, 26). Other previous studies used this duration of fasting to promote feeding in adult rats weighting more than 500 g (6, 7).

### Fasting-Feeding Experiment

Control (*n* = 16) and LP rats (*n* = 16) were fasted for 48 h (water *ad libitum*) and then divided into two subgroups: one group not refed and the second group refed during 2 h. Rats were killed by CO_2_ inhalation and cervical dislocation. Stomach, duodenum, ileum, and nodose ganglia were rapidly dissected and collected for further analysis. Portal blood sample was collected in EDTA-containing tubes (Coveto, Montaigu, France). To preserve active form of gastrointestinal peptides, portal blood (200 µL) was collected in less than 30 s and directly flushed within EDTA tubes containing a mix of protease inhibitors including dipeptidyl peptidase IV inhibitor (diprotin A 100 µM, Sigma, Saint Louis, MO, USA), serine protease inhibitors (aprotinin, approx. 400 TIU/L, Sigma), and protease cocktail inhibitors (diluted 1/100, Sigma). After centrifugation, plasma (50 µL) was then aliquoted in microtube containing the same mix of protease inhibitors and immediately frozen at −80°C.

### Plasma Gastrointestinal Peptides

Plasma concentration of rat non-sulfated CCK-8 (CCK-8NS) and desacylated ghrelin was analyzed by ELISA (EIA kit, Phoenix France, Strasbourg and SpiBio, Montigny le Bretonneux, France, respectively). Plasma total glucose-dependent insulinotropic peptide (GIP) and total PYY were assayed by Milliplex mag (Rat metabolic magnetic bead panel kit, Millipore, MA, USA).

### Kinetics of CCK Release

Rats (*n* = 9–12 animals/group) were fasted for 15 h and refed (chow) at the beginning of the dark phase. This duration of fasting was chosen to induce hunger in adult rats while limiting leptin deficiency that attenuated response to meal-related satiety signals (27). Their FI was measured by food tray weighing at 60, 90, and 150 min post-refeeding. Concomitantly, blood was collected from the tail vein in EDTA-containing tubes (Microvette CB300 EDTA 3K, Sarstedt, Marnay, France) at 0 (15 min before refeeding), 15, 30, 60, 90, and 150 min after the beginning of the meal.

### Sensitivity to CCK

Rats were deprived of food during 15 h and were intraperitoneally injected with either sterile NaCl 0.9% (saline) or CCK octapeptide, sulfated (CCK-8S, Bachem, Germany) just before light off and refeeding. FI and spillage were weighed every 30 min during 90 min. The satiating effect of CCK-8S was tested on distinct animals, for three doses [0.25, 2.5, and 7.5 nmol/kg of bodyweight (BW)] vs vehicle (saline) on consecutive days. It means that each rat received one of the treatment on a given test day followed by a period of 3–5 days of wash out and resting between each injection. Therefore, the whole experiment extended over 3 weeks. Doses of CCK were chosen on the basis of previous published data. The low dose (0.25 nmol/kg BW) reduces 1-h FI by 25% in standard fed rats (28). The 10-fold higher dose (2.5 nmol/kg BW) is necessary to induce FI reduction in diet-induced obesity rats (29) and the higher dose (7.5 nmol/kg BW) is used to induce anorectic effect in MC4R^−/−^ obese rats (30).

### CCK-Vagal Activation

Rats were fasted for 15 h and then received an intraperitoneal (i.p.) injection with either CCK-8S (0.25 nmol/kg BW) or saline 5 min before refeeding and light off. Twenty minutes after injections, rats were killed by CO_2_ inhalation followed by cervical dislocation and nodose ganglia were rapidly collected for Western blot analysis.

### Western Blot Analysis

Nodose ganglia were homogenized at 4°C in RIPA lysis and extraction buffer as previously described (31). Protein concentration was determined using the Bio-Rad protein assay (Bio-Rad, Marnes la Coquette, France). Fifteen micrograms of protein was solubilized in electrophoresis sample buffer, loaded in ready-to-use (4–15%) polyacrylamide gels (Mini-protean TGX, Bio-Rad) and transferred onto Trans-Blot Turbo membrane (Bio-Rad). Protein was probed with anti-pCaMKII mouse polyclonal antibody (diluted 1/2,000, pT286, Thermo Scientific) and anti-CaMKII rabbit monoclonal antibody (diluted 1/1,000, Abcam, France). Following de-hybridization, membrane was probed with rabbit polyclonal anti-pERK and total ERK antibodies (diluted 1/1,000, Abcam). Immunoreactive bands were visualized with DyLight™680- and 800-conjugated antibodies, respectively (KPL, Eurobio, France). Band intensities were quantified by infrared scanning densitometry (Odyssey Imaging Systems, LI-COR, Germany). Data are expressed as the ratio of phosphorylated protein relative to total protein. Protein load was controlled with anti-actin mouse monoclonal antibody (diluted 1/2,000, Sigma).

### Immunohistochemistry

Cryostat sections (7 µm) of fixed nodose ganglia in 4% paraformaldehyde were mounted on SuperFrost Plus Gold slides (Thermo Scientific; Braunschweig, Germany). Sections were stained with a rabbit polyclonal antibody raised against CCK-1R diluted at 1/200 (Santa Cruz Biotechnology, CA, USA). The secondary antibody used at appropriate dilution (1/1,000) was a goat biotinylated anti-rabbit polyclonal antibody (Vector Laboratories, Clinisciences, Nanterre, France) followed by Alexa Fluor^®^ 488-conjugated streptavidin antibody (Molecular Probes, Life Technologies). Nuclei were counterstained with DAPI. Tissues sections were mounted in Prolong Gold antifading medium (Molecular Probes, Thermo Scientific, Courtaboeuf, France). Three to five sections of nodose ganglia per rat were analyzed by fluorescence microscopy (Zeiss Axiovert 200M, Carl Zeiss, France). Fluorescence was quantified on image mosaic representing the total surface of each longitudinal nodose ganglia section using Volocity software (Volocity 6.2.1, Perkin Elmer, France).

### Real-time Quantitative Polymerase Chain Reaction (RT-qPCR)

Total RNA was isolated with TRIzol reagent (Invitrogen, Life Technologies) and treated for 45 min at 37°C with RQ1 DNAse (Promega, Charbonnières-Les-Bains, France). One microgram RNA was reverse-transcribed using Superscript III Reverse Transcriptase (Invitrogen). Five microliters of a 1/20 (nodose ganglia) or 1/40 (intestinal tissue) dilution of cDNA solution were subjected to RT-qPCR in a Bio-Rad iCycler iQ system using the qPCR SYBR Green MasterMix (Fermentas, Courtaboeuf, France). Quantitative PCR consisted of 45 cycles, 30 s at 95°C and 30 s at 60°C each. Primers sequences are figured in Table S2 in Supplementary Material.

### Statistical Analysis

All statistical analyses were carried out using GraphPad Prism 6 (GraphPad Software, Inc., San Diego, CA, USA) software. Comparisons between control and LP groups were performed with an unpaired, two-tailed, Mann–Whitney test. When measures were repeated (dose–response), Friedman test was applied following Wilcoxon signed rank test. A *P* value ≤ 0.05 was considered statistically significant.

## Results

### First-Meal Pattern and Plasma Gastrointestinal Peptides

**Figure 1:**
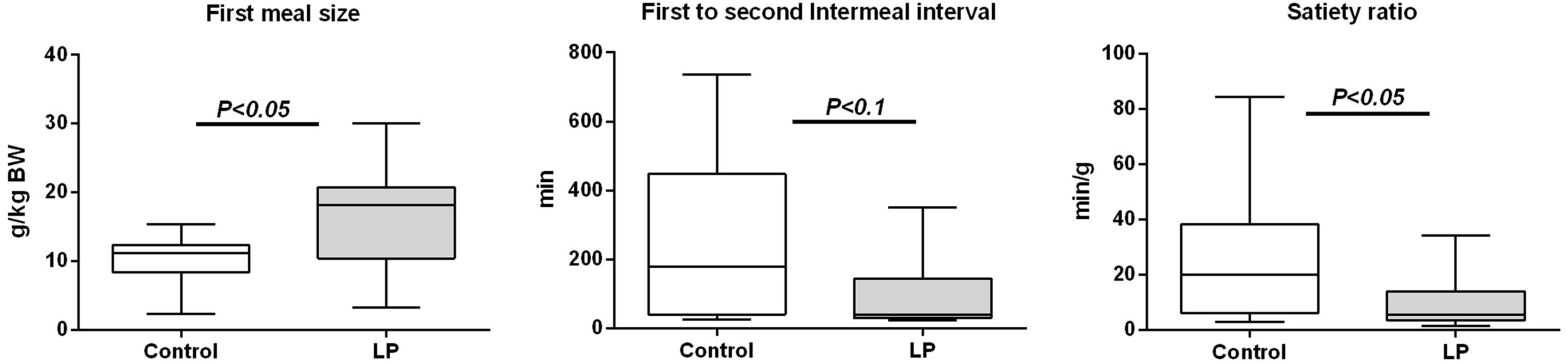
**First-meal pattern measured in physiological cages after 48-h fasting in 160-day-old control [*n* = 12; 631 ± 67 g bodyweight (BW), open bars] and low-protein (LP) (*n* = 10; 551 ± 31 g BW, gray bars) rats (BWs are means ± SD)**. Animals were refed *ad libitum* at 8:00 a.m. and meal pattern was recorded during 24 h. Data are plotted as min to max values in box and whisker with *P* < 0.05 considered as significant difference between the two groups (Mann–Whitney test).

**Figure 2:**
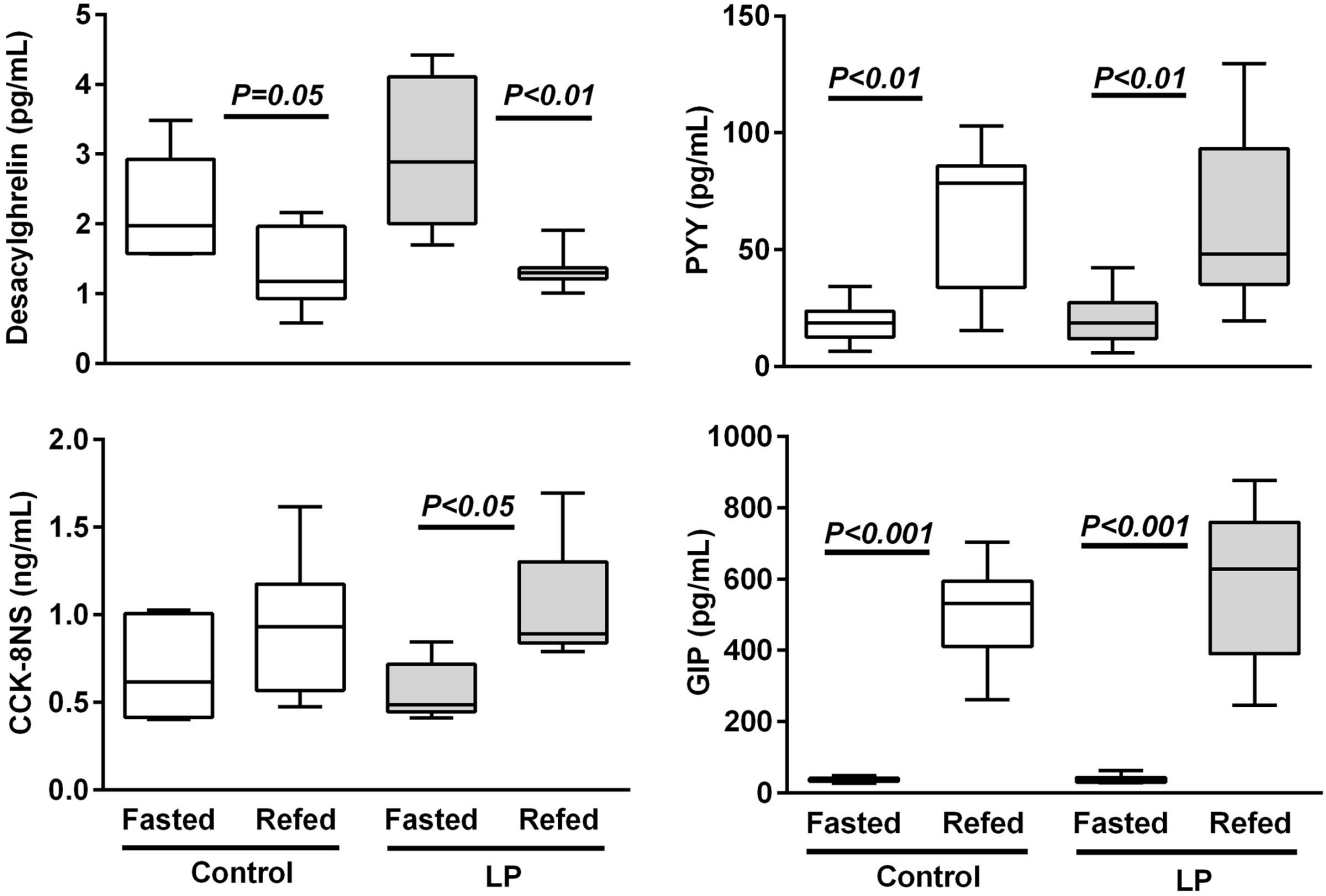
**Plasma gastrointestinal peptides in 48 h-fasted and 2 h-refed 160-day-old control and low-protein (LP) rats (*n* = 8 in each subgroup)**. Data are plotted as min to max values in box and whisker with *P* < 0.05 considered as significant difference between the fasted and refed subgroups (Mann–Whitney test).

Following 48-h fasting, first-meal size (grams per kilogram BW) was significantly higher in LP rats and the inter-meal interval preceding the second meal tended to be reduced, leading to a significant 30%-lower satiety ratio in LP rats as compared to the control group (Figure 1). Since these short-term parameters of FI are primarily regulated by gastrointestinal peptides, gene expression (Figure S1 in Supplementary Material) and plasma concentration of ghrelin (stomach), CCK, GIP (duodenum), and PYY (ileum) were performed in fasted and 2 h-refed control and LP rats (Figure 2). As expected, plasma desacylghrelin was significantly decreased 2 h post-feeding but there were no differences between control and LP rats. Basal plasma concentration of CCK-8NS was similar in the both groups suggesting no IUGR effect on CCK production by I-cells. By contrast, postprandial CCK in LP rats was significantly higher than basal concentration 2 h post-feeding whereas in control rats CCK concentration reached back the initial basal level. This result supports the hypothesis of an alteration in the postprandial feedback regulation of CCK secretion and could be related to failure in short-term mechanisms of FI observed in LP rats (enhanced meal size and reduced satiety ratio). Plasma concentration of GIP and PYY (secreted further distally in the gastrointestinal tract than CCK and GIP) were significantly higher 2 h post-feeding as compared to fasting but with no difference between control and LP groups. Neither basal nor postprandial gene expression of these gastrointestinal peptides was affected by perinatal LP diet (Figure S1 in Supplementary Material).

**Figure 3:**
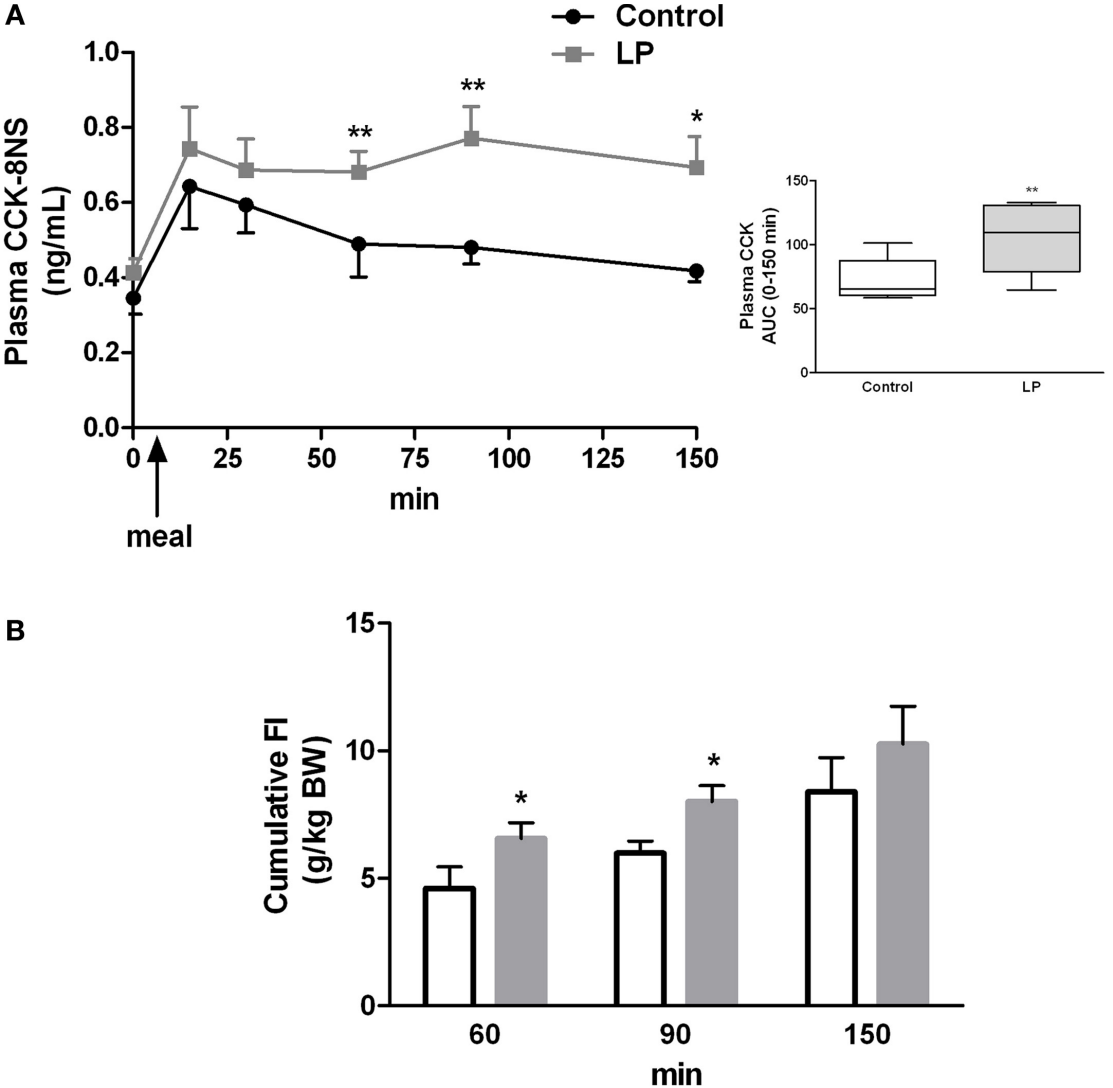
**(A)** Basal and postprandial cholecystokinin (CCK) secretion measured in plasma at different times before and after refeeding by ELISA in control (*n* = 9) and low-protein (LP) rats (*n* = 9). In the box are represented the area under curve (AUC) calculated over the 0- to 150-min period. Rats were fasted 15 h before refeeding at light off indicated by an arrow. **(B)** Cumulative food intake [g/kg bodyweight (BW)] measured by food weighing in the same groups of rats. BWs (mean ± SD) were 658 ± 57 and 621 ± 90 g in control and LP groups, respectively. Values are means ± SEM. Significant differences between control and LP groups at *P < 0.05 and **P < 0.01, respectively (Mann–Whitney test).

Since plasma CCK concentration remained higher than basal concentration 2 h post-feeding in LP rats, we sought for an alteration in the kinetic of production of CCK over the feeding period. Plasma concentration of CCK was measured from 15 min before and until 150 min after feeding. As shown in Figure 3A, CCK release in response to feeding was similar in both groups but from 1 h to 2 h 30 min after FI plasma concentration of CCK was significantly higher in LP rats as compared to control rats. While in control group CCK concentration reached basal value as soon as 60 min post-feeding, values in LP rats were still higher than basal concentration at 150 min post-feeding. The total release of plasma CCK evaluated by the area under curve over the completed period (0–150 min) was significantly higher in LP rats as compared to control rats (Figure 3A). These results clearly showed an alteration in the regulation of postprandial CCK release in LP rats.

Concomitant to higher CCK concentration at 60 and 90 min post-feeding in the LP group, cumulative FI measured for the same period was significantly higher in LP rats than control rats (Figure 3B). This result demonstrated that higher amount of plasma CCK was inefficient to reduce FI in LP rats.

### Sensitivity to CCK-Induced Satiety

**Figure 4:**
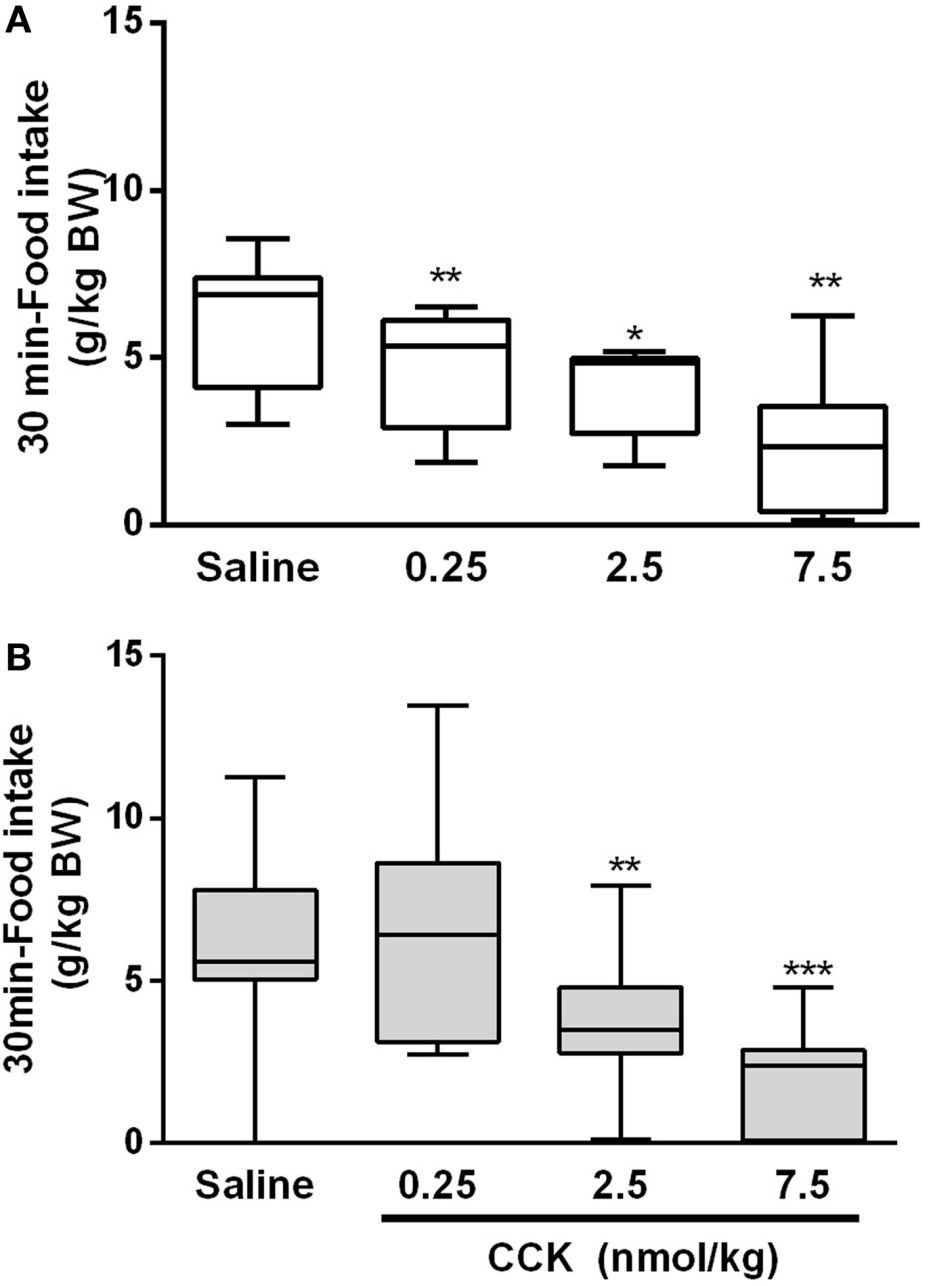
**Food intake during the first 30 min following i.p. injection of CCK and refeeding in control (A) and low-protein (LP) (B) groups**. Bodyweights (mean ± SD) were 655 ± 58 and 590 ± 81 g in control and LP groups, respectively. Each rat received all treatments at different days (repeated measures) with a minimum of 3-day wash out (recovery) between two treatments. *P < 0.05, **P < 0.01 indicated significant differences vs saline group (Friedman and Wilcoxon signed rank tests).

Since CCK is a major intestinal peptide of satiation, we examined its satietogenic effect by measuring FI after an i.p. administration of CCK-8S. As expected, in the control group, CCK induced a dose-dependent decrease of food ingestion in accordance with the normal sensitivity to satietogenic effect of CCK (Figure 4A). The minimal dose of CCK [0.25 nmol/kg; (28)] induced a 25% FI reduction (*P* < 0.01) on the first 30 min as compared to saline in the control group. In LP rats, this minimal dose had no effect on FI. A 10-fold higher dose (2.5 nmol/kg) was necessary to induce a significant reduction of FI in LP rats (*P* < 0.01). This suggested a resistance to the satiety effect of CCK (Figure 4B). At 60 and 90 min post-CCK injection and refeeding, FI was no more significantly different from saline in the both group except for the higher dose of CCK at 60 min (*P* < 0.05) (data not shown).

### Vagal Neurochemical Phenotype and CCK-1R Signaling

**Figure 5:**
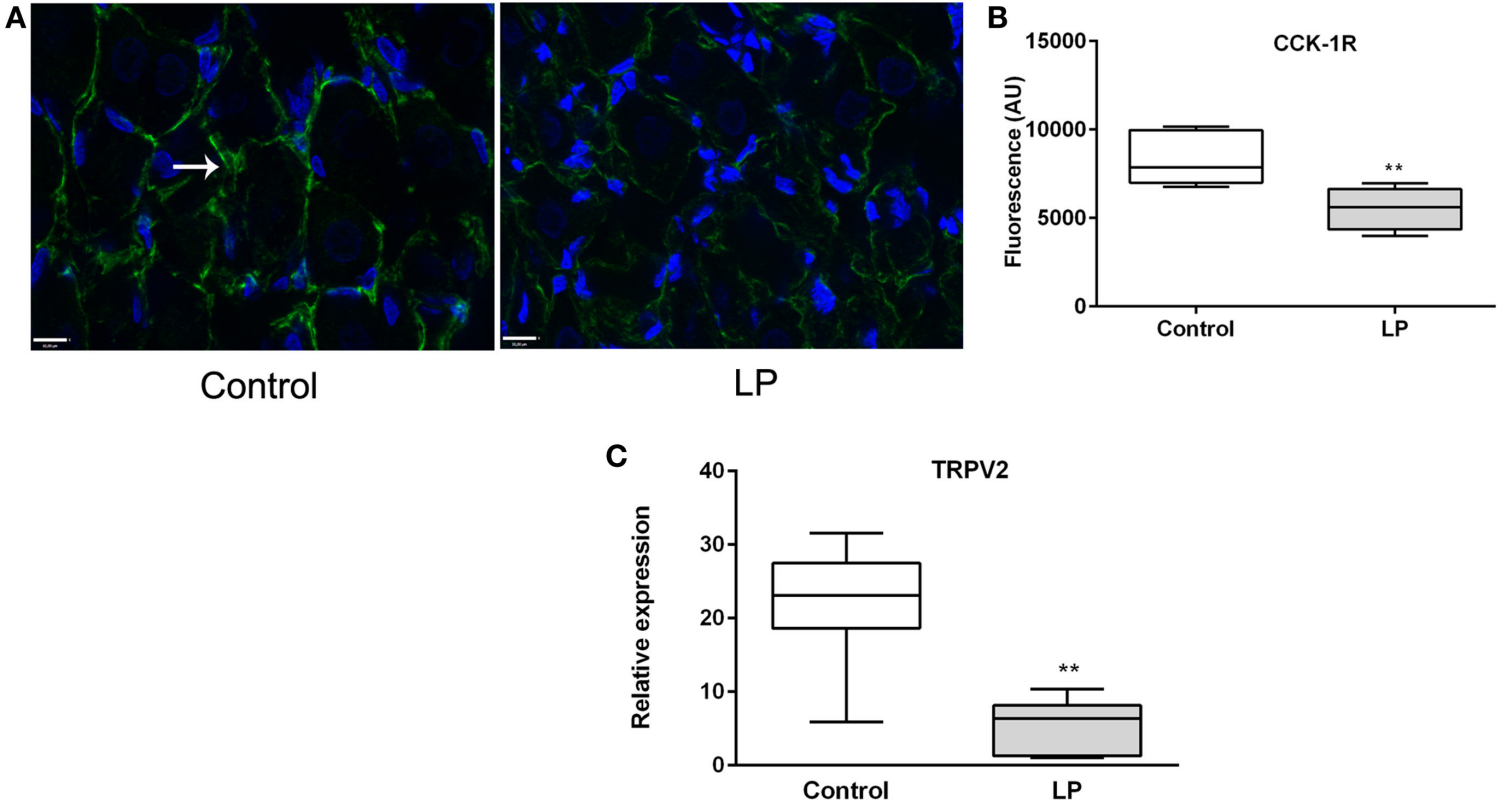
**(A)** Immunofluorescence (×63) of CCK-1R (green) in nodose ganglia of control (left) and low-protein (LP) (right) rats at basal state (fasting). Nuclei are stained in blue with Dapi. **(B)** Corresponding histogram of quantification of fluorescence (*n* = 3–4). **(C)** Relative expression of TRPV2 expression at basal state. Values are means ± SEM. *P < 0.05, **P < 0.01 vs control (Mann–Whitney test).

Since short-term regulation of FI by CCK acts primarily *via* vagal nerve, the expression of CCK-1R was measured in nodose ganglia. The basal expression of vagal CCK-1R measured by immunofluorescence (*P* < 0.01) was lower in LP rats as compared to control rats and could contribute to the loss of sensitivity to CCK-induced satiety in LP rats (Figures 5A,B). No effect of refeeding on vagal CCK-1R expression was observed (data not shown) in the present experiment as previously reported (32). The neurochemical switch operated by CCK on VAN determines an anorexigenic vs orexigenic phenotype that mediates an appropriate response at the brain stem and hypothalamic levels to regulate FI (14, 15). Anorexigenic peptides (CART) and receptors (Y2-R) as well as orexigenic NPY were analyzed in nodose ganglia in response to refeeding in both groups. Unexpectedly, no significant difference was observed in expression of these genes between fasting and refed states, either in control or LP rats (Figure S2 in Supplementary Material).

**Figure 6:**
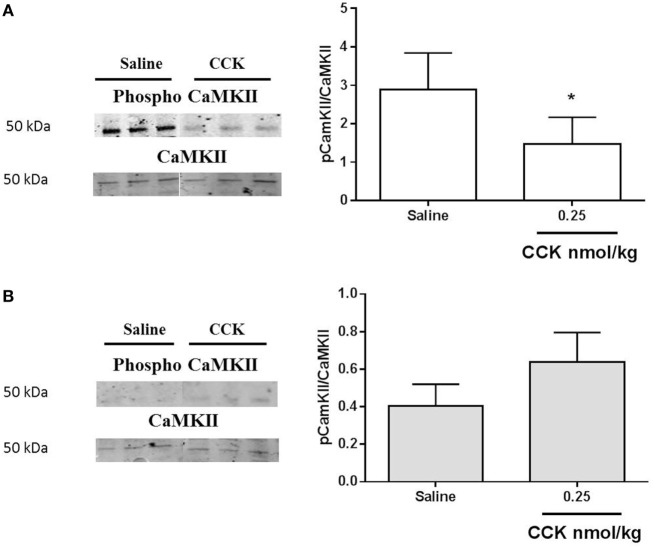
**Western blot analysis of phosphorylated and total calcium/calmodulin-dependent protein kinase (CaMKII) in nodose ganglia of (A) control and (B) low-protein rats in response to i.p. saline or CCK (0.25 nmol/kg bodyweight)**. Values are expressed as the ratio of phosphorylated protein/total protein. *P < 0.05: saline vs CCK (Mann–Whitney test).

Since CCK effects on VAN are mediated by TRPV2–5 cation channels (22), we determined gene expression of TRPV2 and found it significantly under expressed in LP nodose ganglia as compared to control (Figure 5C). Detection of phosphorylated CaMKII has been previously used as a marker of cellular activation in the nodose ganglia (23). Under basal conditions, the ratio pCaMKII/CaMKII was significantly lower in LP nodose ganglia as compared to control (0.4038 ± 0.06 vs 2.888 ± 0.47, respectively, means ± SEM, *P* < 0.05, Mann–Whitney test). Unexpectedly CCK-dependent pCaMKII/CaMKII ratio was reduced 20 min after injection in control rats, with no effect in LP rats (Figure 6).

**Figure 7:**
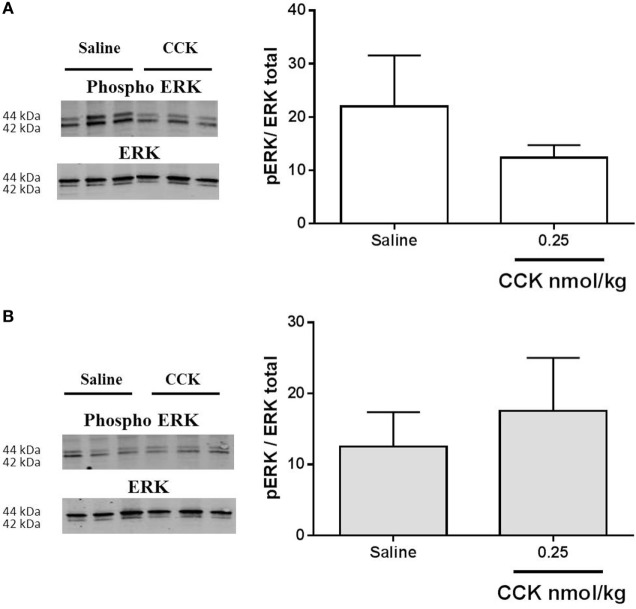
**Western blot analysis of phosphorylated and total ERK1/2 in nodose ganglia of (A) control and (B) low-protein rats (*n* = 4–5) in response to i.p. saline or CCK (0.25 nmol/kg bodyweight)**. Values are expressed as the ratio of phosphorylated protein/total protein. *P < 0.05: saline vs CCK (Mann–Whitney test).

Cholecystokinin activity on CREB phosphorylation in VAN (26) and phosphorylation of ERK in NTS is linked to the satiation effect of the peptide (21, 33). However, 20 min after exogenous administration of CCK, no activation of the ERK pathway was detected in nodose ganglia of control or LP rats (Figure 7).

## Discussion

Although some experimental studies testify alteration of FI and meal pattern in LP rats at adulthood, to the best of our knowledge the present data provide the first evidence that the sensitivity to CCK-induced satiation is impaired by perinatal undernutrition. The resistance to the satiation effect of CCK could be related to a lower expression of CCK-1R and TRPV2 in nodose ganglia in LP rats accredited by a lower basal and phosphorylated level of CaMKII as compared to control rats. The higher postprandial CCK release that we observed in LP rats possibly represents an adaptive mechanism that is partially inefficient at reducing the first meal after a fasting period.

### Short-term FI and Postprandial CCK Are Altered in LP Rats

Alteration of short-term FI has been previously reported in perinatally malnourished rats. Protein restricted diet provided to dams during gestation and/or lactation modify the early appetite of their pups. They demonstrate a hyperphagic phase especially after weaning (7, 34) and during their catch-up growth (6, 7). At adulthood, FI seems grossly normal but a delay in satiation (7) and an increase of the first-meal size following 48-h fasting are still observed suggesting a persistent alteration of short-term regulation of FI. Such an alteration could predispose to obesity particularly when the animals are challenged with a high-calories diet (8). Interestingly, a very recent study showed that LP rats are hyperphagic at older age (1-year old) than younger adult ages usually studied, reinforcing the importance of long lasting impact of programming hyperphagia by perinatal nutrition (35). In the present study using physiological cages that give precise information of meal pattern, we confirmed that adult (160-day old) LP rats present an altered regulation of the first-meal pattern after a long period of fast.

Short-term FI is regulated by a vago-vagal reflex initiated by arrival of food in the stomach and the upper intestine that are sensitive to both distension and nutrients. It ensues digestive secretion and release of appetite-regulating gut peptides (CCK, serotonin, GIP, PYY, glucagon like peptide-1, etc.), all known, at the exception of ghrelin, to reduce meal and delay the next meal (36). We first hypothesized that gene expression as well as the basal and postprandial release of those peptides may be altered in LP rats. A previous study reported elevated plasma ghrelin and reduced CCK and PYY in adult IUGR rats obtained by a 50%-caloric restriction of their dams diet during gestation and maintained restricted during lactation (37). These changes were paralleled to mRNA levels in gastric and intestinal tissues. In this drastic model, as previously shown by others (38), rats are hyperphagic during the whole period of the experiment (from weaning to 7- to 9-month olds). However, only fasting GI peptides concentrations were reported in that study. Therefore, no conclusions on their dynamic change in relation to FI can be drawn. In the present work, offspring obtained by protein restriction of theirs dams during gestation and lactation showed no modification in gene expression of GI peptides, nor at basal or postprandial states. We then measured their plasma concentrations and found that only postprandial CCK was significantly higher than basal in 2-h-refed LP animals. This result seems in contrast with the increased first-meal size and the reduced satiety ratio we measured in LP rats but can be interpreted as if a higher CCK release was inefficient to correctly regulate FI. The kinetic of plasma CCK release post-refeeding was similar in both groups suggesting no delay in CCK secretion by I-cells. By contrast, the feedback regulation seen by the reduced FI 1 h post-refeeding in control rats seemed ineffective in LP rats. As previously mentioned, short-term regulation of FI is initiated by proximal gut mechano- and chemoreceptors, in association with GI peptides release. These initials signals are integrated by VAN to the hindbrain, which triggers a vago-vagal reflex to decrease FI by inhibiting gastric emptying, stimulating digestive secretion, etc. The release of GI peptides is supposed to be regulated by a classic downregulation of theirs receptors once stimuli (nutrients) have moved to distal parts of gut. Thus, the higher postprandial plasma CCK found in LP rats led us to propose the existence of a state of resistance to the satietogenic effect of CCK in these rats.

### LP Rats Are Resistant to CCK-Induced Satiation

Exogenous administration of CCK-8S in LP rats at a dose previously shown to induce satiety in refed rats (28) was not efficient to reduce FI in contrast to control group where the consumption of food was 25% reduced. Since the satietogenic effect of CCK is mediated by CCK-1R on VAN, we hypothesized that perinatal malnutrition could affect CCK signaling *via* its receptor on the vagus nerve. We effectively found that CCK-1R immunoreactivity was significantly lower in nodose ganglia of LP rats compared to control rats probably contributing to the resistance to CCK. A reduced sensitivity to a satietogenic dose of CCK, leading to hyperphagia, has already been shown in obesity-prone rats receiving standard chow (39) as well as in high-fat diet fed rats (40). In diet-induced obese rats, neurochemical analysis of VAN supports a vagal resistance to CCK and leptin in this model in which hyperphagia occurred concomitantly with this resistance (29). In DIO mice, spontaneous activity of VAN innervating the jejunum is weaker as compared to control and the number of afferent neurons that respond to CCK is reduced (41). The impact of perinatal malnutrition on the activity/neurochemical phenotype of the vagus nerve is poorly documented. One study reported a reduced vagal firing rate in adult rats reared by mothers fed a LP diet leading to an impaired efferent vagal activity (42). Electrophysiological studies of nodose neurons of LP rats are in progress in our lab to better characterize the effect of perinatal malnutrition on the activity of the vagus nerve.

Dockray and collaborators have considered CCK as a gatekeeper of the vagal phenotype, switching from an orexigenic phenotype during fasting at low CCK concentration to an anorexigenic one when CCK is released at refeeding (16, 25, 43). Here, we did not observe any modifications of the vagal phenotype between fasting and refed states neither in control nor in LP rats. This discrepancy could be related to different duration of fasting between experiments, even if our conditions (48-h fasting) were close to that of the earlier studies (24–48 h). A very recent study in mice also failed to reproduce this metabolic switch of VAN from an anorectic to orexigenic phenotype (44). Using confocal microscopy to visualize all afferent visceral C-fibers of the vagus nerve, these authors showed that the neuropeptide CART was not regulated by metabolic challenge (fasting or high-fat diet). They also failed to detect any production of MCH, an orexigenic neuropeptide previously shown to be produced by CART neurons in fed state in response to fasting. In our study, we did not detect MCH (mRNA or protein) in nodose ganglia of control and LP rats, whatever they were in their fed or fasted state (data not shown). Thus, our data could not support the hypothesis of an alteration of the vagal phenotype switch by CCK in LP rats but led us to consider CCK signaling in nodose ganglia.

Following its receptor activation, in synergic interaction with leptin receptors, CCK induces a cascade of signal transduction pathways leading to neuronal firing (45). Among them, p-ERK1/2 in terminal endings of VAN in NTS is central to CCK-induced inhibition of FI (19, 21). In the present study, we did not measure any activation of ERK following CCK injection in nodose ganglia. Previous data showing CCK-induced phosphorylation of ERK in NTS reported that the effect occurred very quickly, as soon as 6 min following injection (21). Here, we measured CCK signaling 20 min after stimulation, this may have contributed, in combination with the low dose used, to the absence of phosphorylation signal in nodose ganglia. An analysis on a shorter time needs to be further performed and CCK signaling measured at the afferent endings in the NTS may provide a better comprehension of the underlying mechanism. Concerning calcium signaling, we showed that LP lowered TRPV2 expression in nodose ganglia. This observation could be put in relation with the attenuated level of CaMKII and pCaMKII measured in this model as compared to normal birth-weight rats. Such a reduced expression of CaMKII has been previously reported in the frontal cortex in young adult LP rats (46). Similarly, in LP fetal brain, number of CaMKII immunopositive cells was decreased as compared to control (47). The major role of CaMKII in mediating glutamate signaling has been extensively studied in postsynaptic events implied in memorization and cognition, which are altered in IUGR infants and animal models (48). As proposed by Flores et al., underexpression of CaMKII in frontal cortex of LP rats could be related to altered synaptic plasticity and decreased learning performances in this model. In our study, the reduced expression of CaMKII in nodose ganglia may contribute, together with the reduced CCK-1R expression, to the hyposensitivity of afferent neurons to CCK. Actually, factors leading to this decreased basal expression related to perinatal LP environment are unknown. Epigenetic mechanism is now widely accepted as a memory of antenatal undernutrition exposure throughout life. Research of epigenetic marks on the promoter of the CaMKII gene would be of great interest to link early LP environment and CaMKII expression. More unexpectedly, exogenous CCK induced a decrease in phosphorylation of CaMKII in nodose ganglia of the control group of rats. It has been previously demonstrated in cultured nodose neurons that the CCK-induced increase in cytosolic calcium concentrations is dependent on extracellular calcium influx rather than mobilization on intracellular stores (49). In neurons of the dorsal root ganglia, such a decrease in CaMKII autophosphorylation has been reported *in vitro* by depleting extracellular calcium (50). The significance of this observation in the present study needs further investigation.

In conclusion, we showed for the first time in the present study that adult perinatally undernourished rats have a reduced first-meal satiety ratio associated to a higher postprandial plasmatic CCK release, a reduced sensitivity to CCK when injected at low concentration and a reduced presence of CCK-1R and TRPV2 in nodose ganglia. Altogether, the present data demonstrated a reduced vagal sensitivity to CCK in LP rats at adulthood, which could contribute to deregulation of FI reported in this model.

## Ethics Statement

All experiments were conducted in accordance with the European Union regulations for the care and use of animals for experimental procedures (2010/63/EU). Protocols were approved by the local Committee on the Ethics in Animal Experiments of Pays de la Loire (France) and the French Ministry of Research (Projects 2011.4 and 0271.01). Animal facility is registered by the French Veterinary Department as A44276.

## Author Contributions

GLD designed the study, GLD and CP wrote the protocol, and MN wrote the first draft of the manuscript. MN and CP managed the literature searches and performed animal experiments and analyses. MN performed the statistical analyses. PP contributed for interpretation of data and revised the manuscript. All authors contributed to and have approved the final manuscript.

## Conflict of Interest Statement

The authors declare that the research was conducted in the absence of any commercial or financial relationships that could be construed as a potential conflict of interest.

## Abbreviations

BW, bodyweight; CART, cocaine amphetamine-regulated peptide; CaMKII, calcium/calmodulin-dependent protein kinase; CCK, cholecystokinin; CCK-1R, CCK-1 receptor; ERK, extracellular signal-regulated kinase; FI, food intake; GIP, glucose-dependent insulinotropic peptide; I.P., intraperitoneal; IUGR, intrauterine growth retardation; LP, low protein; MCH, melanin-concentrating hormone; NPY, neuropeptide Y; NTS, nucleus of the solitary tract; PYY, peptide YY; TRPV, vanilloid family of transient receptor potential; VAN, vagal afferent nerve; Y2-R, Y2 receptor.
