# Supplementary material for: Loss of Vagal Sensitivity to Cholecystokinin in Rats Born with Intrauterine Growth Retardation and Consequence on Food Intake
Source: Front Endocrinol (Lausanne). 2017 Apr 10;8:65. doi: 10.3389/fendo.2017.00065 (PMC5385335; doi:10.3389/fendo.2017.00065)
Supplement: Supplementary file 1 [file Table_1.DOCX]

**Table S1**. Composition of experimental diets (g/kg diet)

| Macronutrient | Control  (20 % protein) | Low protein  (8 % protein) | Standard Chow |  |
| --- | --- | --- | --- | --- |
| Casein  Corn starch  Maltodextrin 10  Vegetable oil  Cellulose  Vitamin Mix  Mineral Mix  **Energy density (kcal/g)**  **% macronutrient kcal/g**  Protein  Carbohydrate  Fat | 220  80  552  43  50  10  47  3.7  20  63  4.3 | 90  80  682  43  50  10  48  3.7  8.0  76  4.3 | 180  630  37  53  10  40  3.6  16  64  3 |  |
