# Supplementary material for: Loss of Vagal Sensitivity to Cholecystokinin in Rats Born with Intrauterine Growth Retardation and Consequence on Food Intake
Source: Front Endocrinol (Lausanne). 2017 Apr 10;8:65. doi: 10.3389/fendo.2017.00065 (PMC5385335; doi:10.3389/fendo.2017.00065)
Supplement: Supplementary file 2 [file Table_2.DOCX]

**Table S2.** Primer sequences used for analyses of mRNA expression.

| Gene | GeneBank  Accession | Forward primer | Reverse primer |
| --- | --- | --- | --- |
| β actin | [NM_031144.3](https://www.ncbi.nlm.nih.gov/nuccore/NM_031144.3) | CTATCGGCAATGAGCGGTTCC | GCACTGTGTTGGCATAGAGGTC |
| Cartpt | NM_017110.1 | CCTACTGCTGCTGCTACCTTTG | CCGAAGTTGCCGCCTTGG |
| Cck | NM_012829.2 | TGCTTGGAGGAGGCGGAATG | GCTGGGCTGAGGTGTGTGG |
| Cckar | NM_012688.3 | TCCTTCATCCTCCTCCTCTCCTAC | CCTCCCATCCTCCTCCTCTCC |
| Gapdh | [NM_017008.4](https://www.ncbi.nlm.nih.gov/nuccore/NM_017008.4) | CGGCAAGTTCAACGGCACAG | TCCACGACATACTCAGCACCA |
| Ghrl | NM_021669.2 | AGAGGCGCCAGCTAACAAGTAA | GCAGGAGAGTGCTGGGAGTT |
| Gip | NM_019630.3 | CTCCTGTTCCTGGCTGTC | GGCGATGCTGTAATCACTG |
| Pyy | NM_001034080.1 | AGCGGTATGGGAAAAGAGAAGTC | ACCACTGGTCCACACCTTCTG |
| Npy | NM_012614.2 | GTGGACTGACCCTCGCTCTATC | ATGAGATTGATGTAGCGCAGA |
| Npy2r | NM_023968.1 | TTTCCACCCTGCTAATCCTCTACG | TTGTGCCTTCGCTGATGGTAATG |
| TRPV2 | [NM_001270797.1](https://www.ncbi.nlm.nih.gov/nuccore/NM_001270797.1) | ACAGCCCCATTACTGTCAGC | CAGGCCTGGCTGGGAC |

Official symbol of corresponding genes are listed. Official full name are developed below with current name used in the present study into brackets:

Cartpt: CART prepropeptide; Cckar: cholecystokinin A receptor (= CCK-1R); Ghrl: ghrelin and obestatin prepropeptide; Gip: gastric inhibitory polypeptide (also named glucose-dependent insulinotropic peptide); Npy: neuropeptide Y; Pyy: peptide YY; Npy2r: neuropeptide Y receptor Y2 (= Y2R)
