# Supplementary material for: Loss of Vagal Sensitivity to Cholecystokinin in Rats Born with Intrauterine Growth Retardation and Consequence on Food Intake
Source: Front Endocrinol (Lausanne). 2017 Apr 10;8:65. doi: 10.3389/fendo.2017.00065 (PMC5385335; doi:10.3389/fendo.2017.00065)
Supplement: Supplementary file 3 [file Presentation_1.PPTX]

## Slide 1
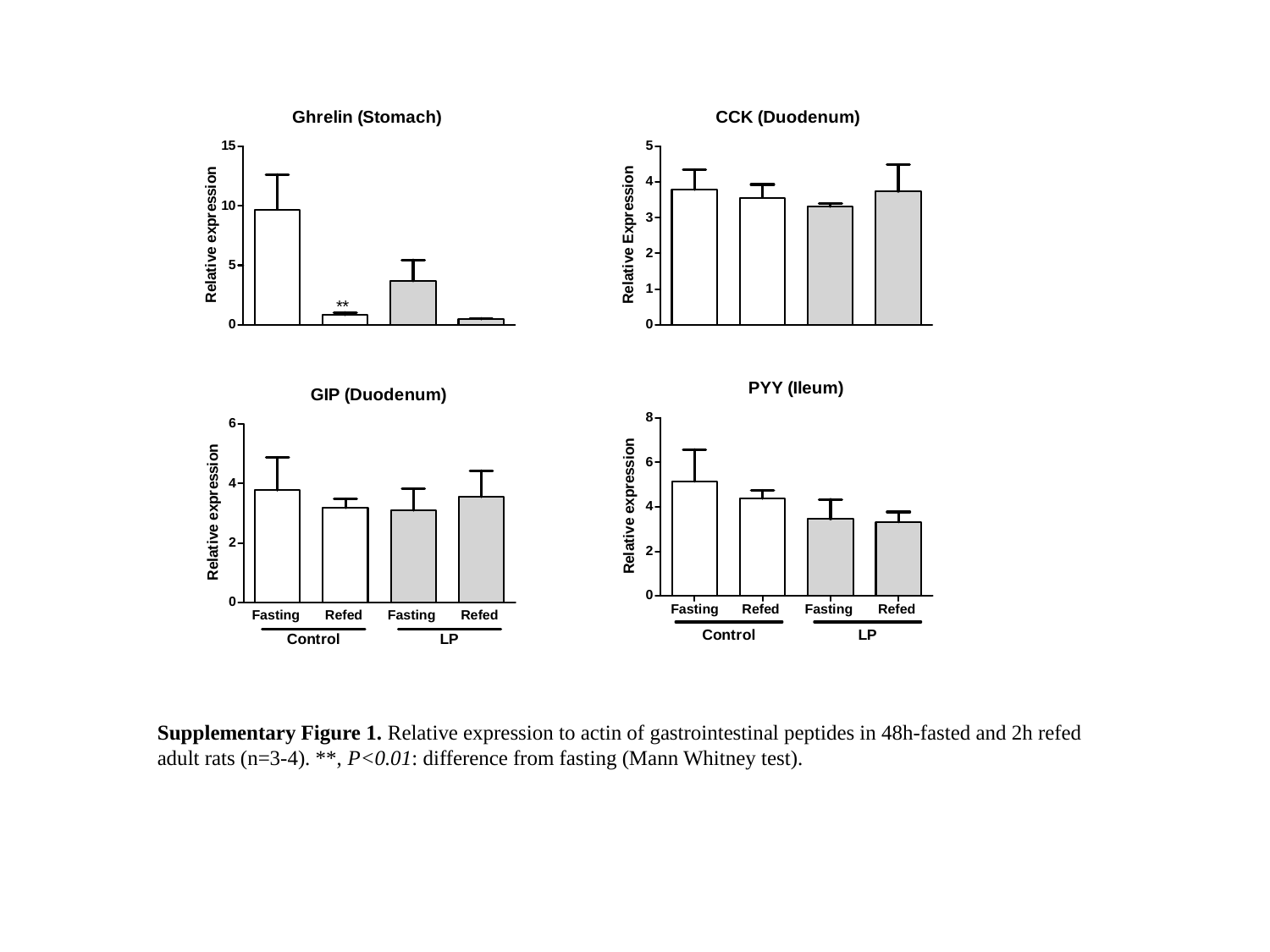

Supplementary Figure 1. Relative expression to actin of gastrointestinal peptides in 48h-fasted and 2h refed adult rats (n=3-4). **, P<0.01: difference from fasting (Mann Whitney test).
