# Supplementary material for: Loss of Vagal Sensitivity to Cholecystokinin in Rats Born with Intrauterine Growth Retardation and Consequence on Food Intake
Source: Front Endocrinol (Lausanne). 2017 Apr 10;8:65. doi: 10.3389/fendo.2017.00065 (PMC5385335; doi:10.3389/fendo.2017.00065)
Supplement: Supplementary file 4 [file Presentation_2.PPTX]

## Slide 1
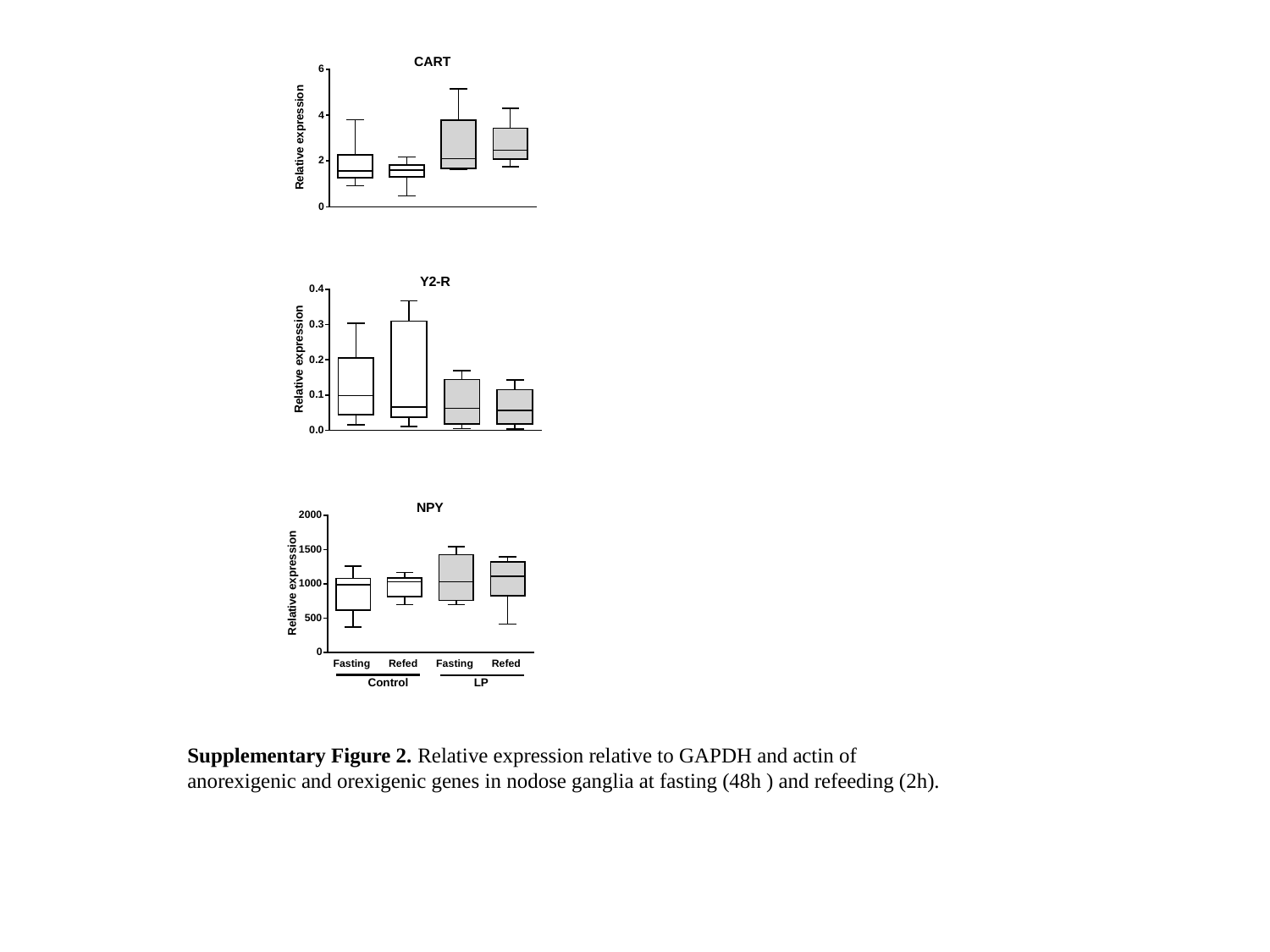

Supplementary Figure 2. Relative expression relative to GAPDH and actin of anorexigenic and orexigenic genes in nodose ganglia at fasting (48h ) and refeeding (2h).
